# *Bothrops* (Fer-de-lance) snakebites in the French departments of the Americas (Martinique and Guyana): Clinical and experimental studies and treatment by immunotherapy

**DOI:** 10.1371/journal.pntd.0011083

**Published:** 2023-02-28

**Authors:** Dabor Resiere, Hatem Kallel, Jonathan Florentin, Stephanie Houcke, Hossein Mehdaoui, José María Gutiérrez, Remi Neviere

**Affiliations:** 1 Cardiovascular Research Team EA7525, Université des Antilles, Fort de France, France; 2 Department of Critical Care Medicine, Toxicology and Emergency, CHU Martinique (University Hospital of Martinique), Fort-de-France, France; 3 Intensive Care Unit, Cayenne General Hospital, Cayenne, French Guiana, France; 4 Instituto Clodomiro Picado, Facultad de Microbiología, Universidad de Costa Rica, San José, Costa Rica; Instituto Butantan, BRAZIL

## Abstract

Snakebite envenomation is a relevant medical hazard in French Guiana and Martinique, two French territories in the Americas. All snakebite envenomations in Martinique are inflicted by the endemic viperid species *Bothrops lanceolatus*, whereas *Bothrops atrox* is responsible for the majority of snakebites in French Guiana, although other venomous snake species also occur in this South American territory. This review summarizes some of the key aspects of the natural history of these species, as well as of their venom composition, the main clinical manifestations of envenomations, and their treatment by antivenoms. *B*. *atrox* venom induces the typical set of clinical manifestations characteristic of *Bothrops* sp. venoms, i.e., local tissue damage and systemic alterations associated with coagulopathies, hemorrhage, hemodynamic alterations, and acute kidney injury. In the case of *B*. *lanceolatus* venom, in addition to some typical features of bothropic envenomation, a unique and severe thrombotic effect occurs in some patients. The pathogenesis of this effect remains unknown but may be related to the action of venom components and inflammatory mediators on endothelial cells in the vasculature. A monospecific antivenom has been successfully used in Martinique to treat envenomations by *B*. *lanceolatus*. In the case of French Guiana, a polyvalent antivenom has been used for some years, but it is necessary to assess the preclinical and clinical efficacy against viperid venoms in this country of other antivenoms manufactured in the Americas.

## Introduction

**Fig 1:**
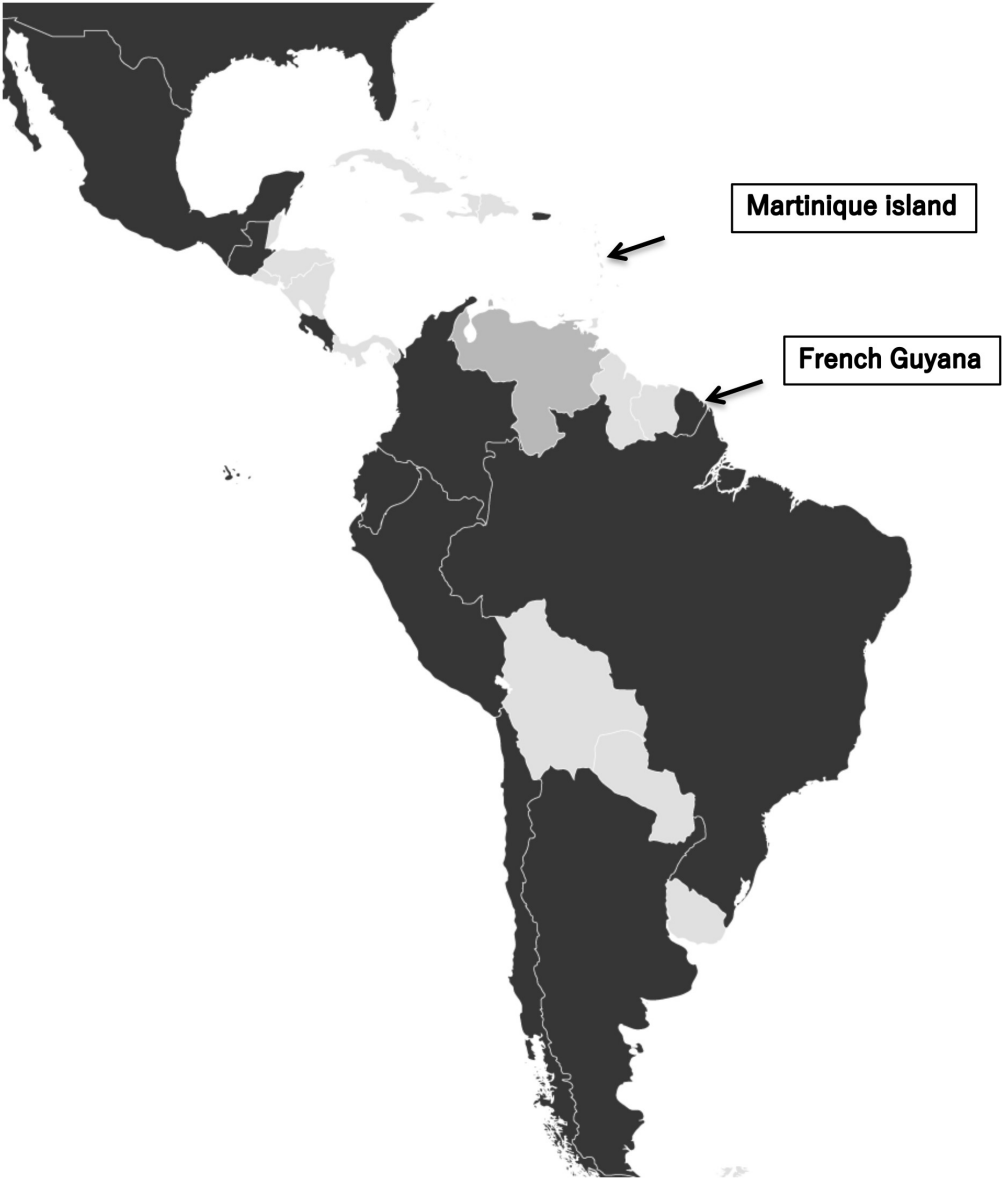
French overseas territories of French Guyana on the northern Atlantic coast of South America in the Guyanas) and Martinique Island in the Caribbean sea.

Overseas France includes island territories in the Atlantic, Pacific Indian Oceans and peri-Antarctic region, and French Guiana in South America. Among these territories, French Guiana and Martinique, a French Lesser Antilles island in the Caribbean Sea, are habitats for many animal species, including hundreds of snake species (Fig 1). In French Guiana and Martinique, *Bothrops* species are responsible for the vast majority of snakebite envenomations [1–3]. Among the hundred species of snakes identified in French Guiana, most cases of snakebites are caused by snakes of the genus *Bothrops*, accounting for 80% to 90% of all snakebite cases, mainly from *Bothrops atrox* [2]. In Martinique, *Bothrops lanceolatus*, a venomous pit-viper species, is endemic to this French Caribbean island [3]. Hence, *B*. *atrox* and *B*. *lanceolatus* may be considered the species responsible for most medically significant snakebite envenomations in these French overseas territories of the Americas.

## 1. *Bothrops atrox* and *Bothrops lanceolatus* snakes natural history

*B*. *atrox* is the most abundant venomous snake in French Guiana [2]. There is no integrative systematic review available, and its taxonomic status remains controversial based on limited animal sampling, which provided genetic evidence that *B*. *atrox* probably represents a complex of several lineages across the species distribution [4]. The non-monophyly of *B*. *atrox* mitochondrial DNA lineages was recently confirmed by other studies based on a more comprehensive geographic sampling [5]. Considering the current taxonomy, *B*. *atrox* is mostly endemic to the Amazon rainforest [6]. The species is predominantly nocturnal, presenting a higher encounter rate at night but may also be active during the day [7]. In French Guiana, *B*. *atrox* inhabits forested areas, although it may be found in disturbed habitats around human settlements, including pastures and crops, and urban areas [8]. *B*. *atrox* is especially active at night when adults are often seen on the ground hunting, while juveniles frequently hunt in vegetation (up to heights of 1.5 m) [7]. Regarding food preferences, this snake is a generalist, feeding on centipedes, fish, amphibians, lizards, other snakes, rodents, marsupials, and birds [9]. Juveniles prefer ectothermic animals (frogs, lizards, and centipedes), and adults prefer endothermic animals, mostly rodents [9].

*B*. *lanceolatus* is the only venomous snake in Martinique [3]. This species, together with *B*. *caribbaeus*, endemic to the neighboring island of Saint Lucia [5], originated as a consequence of the long-distance dispersal of the South American mainland species of the complex *B*. *asper/B*. *atrox*, with which they form a sister clade [5]. *B*. *lanceolatus* and *B*. *caribbaeus* form a monophyletic group divergent at the molecular level from all other South American species of *Bothrops*. It is estimated that the timing of the split of these Lesser Caribbean species from the *B*. *asper/B*. *atrox* clade occurred during the late Miocene or early Pliocene 4.2 to 8.9 million years ago [5]. The geographical distribution of this species in Martinique seems to be disjunct, with two isolated populations confined to humid upland regions in the northern and southern portions of the island [10]. *B*. *lanceolatus* is an inhabitant of tropical moist forest and tropical wet forest, primarily in upland regions. It also occupies rocky hillsides and tends to be arboreal, having been found as high as 20 m above ground [10]. When young, *B*. *lanceolatus* feeds chiefly on lizards, later on birds, and when adults, mostly on rats. In the dense woods, it lies quiet, seldom disturbed but by the singing of some birds that live in the wilderness. The night is the time of its wandering, and it has been seen in the roads crossed by people during the day [10]. *B*. *lanceolatus* has most recently been assessed for The IUCN Red List of Threatened Species in 2015 [11].

## 2. *Bothrops atrox* and *Bothrops lanceolatus* venoms

Over the past decades, proteomic studies of viperid snake venoms, including those of *Bothrops* sp., indicate that the predominant components playing a role in toxicity are zinc-dependent metalloproteinases (SVMPS), phospholipases A_2_ (PLA_2_s), and serine proteinases. Other components include vasoactive peptides, disintegrins, L-amino acid oxidases, cysteine-rich secretory proteins (CRISPs), and C-type lectin-like components [12].

### 2.1 *Bothrops atrox venom*

There are important regional differences in the composition of the venoms of *B*. *atrox* along the Amazon, which are likely to have implications in the pathophysiology and clinical manifestations of envenomations [13]. Indeed, *B*. *atrox* venom variability occurs among wild specimens from different geographical areas and different habitats, life span of individuals (juvenile versus adults), body size, and gender [14]. Venom properties vary ontogenetically in *Bothrops* species of the same genus, likely caused by differences in the feeding habits of juveniles and adults [15]. A study has compared the clinical, epidemiological, and laboratory aspects of envenomation to the size of *B*. *atrox* snakes, suggesting that small snakes (juveniles) are associated primarily with moderate local pathology and with systemic hemorrhagic and coagulopathic manifestations. In contrast, larger snakes were responsible for severe cases and characterized by major local effects (necrosis, edema, phlyctenae, compartment syndrome, and infection) [16].

An initial study of the transcriptome of a young male adult specimen of *B*. *atrox* captured in the Amazonas State of Brazil and maintained in captivity revealed that the major classes of transcripts expressed in venom gland are those of class III metalloproteinases and PLA_2_ [17]. Proteomic and other types of biochemical and functional studies have shown variations in the composition and actions of the venoms of *B*. *atrox* from specimens collected in different localities in South America [13–23]. Proteomics has been extensively used to understand *B*. *atrox* venom composition, from Colombia, Brazil, Ecuador, and Peru [13,21–23] (Table 1). Adult venoms south of the Amazon contain a higher proportion of PIII SVMPs, whereas adult venoms from northern regions contain higher amounts of PI SVMPs and PLA_2_s [21–23].

**Table 1:** Relative abundance of different protein families in the venom proteomes of some populations of *B*. *atrox* (data from [13,23]).

Venom component	Colombia Meta [23]	Colombia Magdalena [13]	Venezuela Puerto Ayacucho [13]	Brazil Amazonas [23]	Brazil Presidente Figueiredo Amazonas [13]	Brazil Monte Alegre Para [13]
PIII metalloproteinase	3.1%	4.8%	54.0	49.1%	68.0%	69.0%
PI metalloproteinase	45.4%	21.0%	31.0	23.0%	16.0%	10.5%
Phospholipase A2	24.1%	48.0%	7.7	14.3%	8.0%	12.8%
Serine proteinase	10.9%	19.0%	2.3	4.6%	4.3%	2.6%
CRISPs	2.6%	2.1	3.8	1.8%	2.2%	3.6%
L-amino acid oxidase (LAAO)	4.7%	2.0	1.2	2.1%	1.5%	1.5%
C-type lectin-like protein	7.1	-	-	0.7%	-	-
Disintegrin	1.7	3.1	-	<0.1	-	-

Notably, proteomic characterization of venom of *B*. *atrox* endemic of French Guiana is lacking. An approximation could be done based on the study of the proteome of the venom of this species from the locality of El Paují (Venezuela) since this location is close to the Territory of French Guiana. This venom has a predominance of PI and PIII SVMPs over other protein families [13] (Table 2).

**Table 2:** Relative abundance of different protein families in the venom proteomes of two populations of *B*. *atrox* and *B*. *lanceolatus* (data from [13,38]).

Venom component	*B*. *atrox* (Sao Gabriel, Brazil) [13]	*B*. *atrox* (El Paují, Venezuela) [13]	*B*. *lanceolatus* (Martinique) [38]
PIII metalloproteinase	66.0%	20.0%	48.4%
PI metalloproteinase	18%	65%	25.8%
Phospholipase A2	8.5%	8.5%	8.6%
Serine proteinase	2.5%	2.2%	14.4%
CRISPs	2.7%	2.8%	-
L-amino acid oxidase (LAAO)	1.4%	1.5%	2.8%
C-type lectin-like protein	0.9%	-	-
Disintegrin	-	-	-

Hemorrhagic syndrome and its pathophysiological consequences remains the most important cause of lethality associated with *B*. *atrox* envenomation [24,25]. Since early studies on biochemical characterization of venom components, *B*. *atrox* venom is known as a rich source of thrombin-like enzymes, classified as Snake Venom Serine Proteinases (SVSPs), which directly hydrolyze fibrinogen, generating feeble fibrin clots [26]. Adding to thrombin-like enzymes, disturbances in hemostasis are also evoked by some toxins in the SVMPs group. The procoagulant mechanism of SVMPs is the direct activation of factors II and X, resulting in the formation of endogenous thrombin [9,24,27]. *B*. *atrox* venom hemorrhagic toxins are well characterized and are involved in the local and systemic effects of human victims of snakebite [28–31]. Thromboelastographic and thromboelastometric studies showing the action of *Bothrops* venoms on human plasma and fibrinogen have recently deciphered coagulotoxic effects of these venoms [18,32]. Significant procoagulant plasma potency was observed with *B*. *atrox* venom [18,32].

Hemorrhage is primarily attributed to PI- and PIII-class SVMPs that induce vascular lesions. The different degrees of hemorrhagic activity of SVMPs have been attributed to their hydrolytic activity of extracellular matrix components such as laminin and type IV collagen, and also to the fact that PIII-class SVMPs bind to targets present on the basement membrane, allowing the concentration of the enzyme adjacent to the capillaries and venules, making more productive the hydrolysis and rupture of the vessel [29,33]. The disruptive action of hemorrhagic SVMPs is potentiated by the alterations in hemostasis induced by procoagulant and thrombin-like enzymes. Together with these procoagulant and hemorrhagic enzymes, *B*. *atrox* venom PLA_2_s also contribute to local tissue damage due to their myotoxic activity, causing lysis of muscle cells and release of intracellular proteins that also enhance the reactive proinflammatory status of the tissues close to the bite [34]. In addition, *B*. *atrox* venom contains toxins that directly induce inflammatory effects, leading to plasma exudation and accumulation of polymorphonuclear and mononuclear leukocytes and macrophages to the injury site [35,36].

In line with the well-known hemostatic disturbances, *B*. *atrox* venoms can elicit coagulotoxic effects in prey captured by snakes, in which hemorrhage, plasma extravasation, and the rapid formation of endogenous thrombin can result in prey incapacitation through circulatory shock or intravascular thrombosis. It is clear that, in the evolution of the venom of *B*. *atrox*, there has been a strong selection for generating coagulotoxic proteins. At the same time, the venom lacks neurotoxic and cardiotoxic components. *B*. *atrox* venom is weakly lethal in mice, with relatively high Median Lethal Dose (LD_50_). The LD_50_ of *B*. *atrox* venom from French Guiana in mice is 131 μg/20 g body weight [37]. In human accidents, the lethality rates are also relatively low, although fatalities may occur in severe cases; in addition, the venom often induces coagulopathies, hemorrhage, and extensive tissue damage [9].

### 2.2. *Bothrops lanceolatus* venom

*B*. *lanceolatus* venom contains a variety of toxins, which belong to SVMPs, SVSPs, and PLA_2_, and L-amino acid oxidases, among others [38] (Table 2). Bites from *B*. *lanceolatus* induce local tissue damage similar to that of *B*. *atrox* but infrequently elicit systemic bleeding and incoagulability. In sharp contrast, *B*. *lanceolatus* envenomations may be complicated by multiple systemic thrombotic events within 48 h after the bite [3]. SVMPs, which induce local hemorrhage and edema formation, are the most abundant components of this venom [38]. Myotoxic PLA_2_s are, however, less abundant in *B*. *lanceolatus* venom mixture. In line with this proteomic profile, this venom induces hemorrhage, whereas the content of PLA_2_s is low, and PLA_2_-induced myotoxic activity is relatively weak, especially compared to other *Bothrops* species [38]. As compared to other snake venoms, low *B*. *lanceolatus* venom PLA_2_ activity has been previously found when determined in the absence of detergent with egg yolk as substrate. Activation of PLA_2_ from *B*. *lanceolatus* venom has been attributed to the catalytic property of the enzyme itself rather than the dissociation of PLA_2_ inhibitors such as in the cases of other *Bothrops* species [39]. Deleterious effects such as hemolytic activity, formation of edema, and vascular permeability increase have been consistenly attributed to bioactive components of *B*. *lanceolatus* venom [40–42].

*B*. *lanceolatus* venom largely lacks in vivo defibrinogenating activity [38,43]. In vitro, *B*. *lanceolatus* venom can clot purified human fibrinogen, indicating the presence of a thrombin-like enzyme [44], but appears to be devoid of defibrinating activity after intravenous injection in mice [38,43]. The basis for the in vivo thrombotic action of this venom remains elusive and does not seem to be due to a procoagulant action of the venom on plasma [38,43]. While venom of *B*. *caribbaeus* can induce platelet aggregation and agglutination in vitro and profound thrombocytopenia in vivo [45], *B*. *lanceolatus* venom induces mild thrombocytopenia [46]. Unlike *B*. *atrox*, *B*. *lanceolatus* venom is not coagulant when tested on human citrated plasma, which may indicate an absence of prothrombin or factor X activators [38,43]. However, recent thromboelastographic studies have reevaluated the procoagulant toxicity trait of venoms of the *Bothrops* genus [18,32]. Using this methodological approach, *B*. *lanceolatus* venom was procoagulant in recalcified and phospholipid-enriched plasma [18].

The mechanisms behind the thrombotic effect induced by *B*. *lanceolatus* venom are unknown. Proposed mechanisms include direct vascular endothelium injury by venom toxins and vascular endothelial cell activation related to inflammatory processes. While *B*. *lanceolatus* seems to exert a weak direct endothelial cell toxicity, endothelial activation/dysfunction can induce thrombosis via activation of the inflammatory response [40,47–50]. Activation of vascular endothelial cells by *B*. *lanceolatus* venom is supported by studies showing increased production of inflammatory cytokines and chemokines, as well as tissue factor expression [47]. *B*. *lanceolatus* venom can elicit strong complement activation leading to high C3a, C4a, and C5a anaphylatoxins levels [47,48]. Complement activation induced by this venom is further accompanied by an intense lipid mediators’ release, which includes LTB4, PGE2, and TXB2 along with cytokine (IL-1β, IL-6, and TNF-α) production and chemokine (CCL2, CCL5, and CXCL8) and osteopontin up-regulation [48–50]. These outcomes show that *B*. *lanceolatus* venom causes a strong inflammatory reaction in the blood, which might favor thrombus formation by inducing a procoagulant phenotype in endothelial cells.

## 3. Envenomation in humans

### 3.1. *B*. *atrox* envenomation in French Guiana

In South America, epidemiological studies suggest that the majority of snakebite cases are caused by different species of snakes of the genus *Bothrops*. A study carried out in the Amazonian state of Rondônia, Brazil, a region with a high incidence of snakebites, showed that most victims are adult men (20 to 50 years) living in rural areas of the Brazilian agricultural frontier, who are bitten during work activities [51]. A higher incidence of envenomations occurred during the rainy season and during the diurnal period of the day, while most venomous snakes have nocturnal habits [51,52]. Likewise, *B*. *atrox* is responsible for the majority of snakebites in French Guiana [2].

Clinical features of *B*. *atrox* envenomation in French Guiana resemble the classic syndrome associated with bites from viperids, including local effects at the bite site (pain, edema, blistering, bleeding, necrosis) and systemic effects (hypotension, coagulopathies, systemic hemorrhages) [2,8,53–55]. The venoms of snakes of the genus *Bothrops* are hemorrhagic, damaging the vascular endothelium and consuming coagulation factors, i.e., inducing venom-induced consumption coagulopathy, and eliciting inflammation [55–58]. Blister formation typically occurs following bites by *B*. *atrox* and has been related to inflammatory processes and poor local prognosis as they increase the risk of infection and necrosis [59]. Complications of *B*. *atrox* envenomation may also include compartment syndrome of the bitten muscle, wound infection, hemodynamic instability and shock, and acute kidney injury (AKI) [2].

Though the precise number of snakebites is unknown, an estimated annual incidence of envenomation in French Guiana exceeded 20 cases per 100,000 inhabitants, with a case fatality rate of 3% [1]. From 2007 to 2015, 283 patients with snakebite envenomations were admitted to Cayenne General Hospital. Among them, 43 (15.8%) were considered severe, and 4 ultimately led to death (1.4% case fatality rate in people suffering real envenomations) [60,61]. From 2016 to 2019, similar results were observed in a prospective observational study conducted on 133 patients admitted to the Intensive Care Unit (ICU) of Cayenne General Hospital for snakebite envenomation [2]. In this work, envenomation was considered severe in 26.2% of cases. Snake identification was performed in 51% of cases, and *B*. *atrox* was identified in 43.6% of cases. The main clinical manifestations were edema (98.5%), pain (97.7%), systemic hemorrhage (18%), blisters (14.3%), and local hemorrhage (14.3%). Circulatory shock (1.5%), AKI (15%), and systemic hemorrhage (18%) were the most frequent complications. The elapsed time from snakebite to the development of systemic hemorrhage was 4.5 h. Infections such as abscesses (65%), necrotizing fasciitis (18.6%), and cellulitis (27.9%) were recorded in this group of the patients. In this series, overall mortality was 1.5% [2].

AKI is the main systemic complication and a common cause of death in *Bothrops* envenomation [62]. Several epidemiological studies describe a great variation in AKI incidence ranging from 6% in *B*. *atrox* to 38.5% in *B*. *asper* [50]. Proposed mechanisms of *Bothrops* venom-induced AKI include the direct action of venom on the kidney, the hemodynamic alterations, myoglobinuria, hemoglobinuria, glomerular microthrombi deposit due to coagulation abnormalities, and immunologic mechanisms [62–64]. In experimental models using viperid snake species, kidney hemodynamic changes varied according to the snake species but typically presented renal vascular resistance and glomerular filtration rate decreases [65]. Overall, a multifactorial origin of AKI is proposed, which emphasizes the role of hemodynamic disorders with bleeding or fibrin deposits in tubular structures, inflammatory processes, formation of immune complexes, and nephrotoxic action of venom.

Administration of antivenom is the only specific treatment to counteract *Bothrops* snakebite envenomation [12,66]. Antivenom typically comprises concentrated immunoglobulins, which are refined to produce F(ab′)_2_ fragments, although some antivenoms are made of whole IgG molecules. There are two main types of antivenom, namely monospecific (or monovalent) antivenom, which is generated against the venom of a single snake species, and polyspecific (or polyvalent) antivenom, which is made using the venom of multiple snake species as immunogens [66].

In French Guyana, *Bothrops* envenomations are treated with Antivipmyn-Tri, a polyvalent antivenom for *Bothrops* and other viperid species (Instituto Bioclon, Mexico) [53,67]. This antivenom neutralizes the lethal, hemorrhagic, and in vitro coagulant activities of venoms of *B*. *atrox* endemic of French Guiana [68]. In this study, the intravenous route of injection was used to assess lethality. Since 2014, Antivipmyn-Tri has been used in this country, and preliminary preclinical and clinical reports of its efficacy and safety in envenomings by various species have been presented [2,53,68–70]. Retrospective studies that evaluated the efficacy in severe *Bothrops* envenomations in French Guiana (Saint-Laurent-du-Maroni, Western Guiana Hospital, and Cayenne General Hospital) failed to demonstrate evidence of clinical benefit [53]. However, another study showed that the time from *Bothrops* snakebite to achieve normal dosages of coagulation parameters was shorter in patients who received antivenom than in those who did not [2]. These conflicting findings urge for more detailed evaluations and also for the need to consider the possibility of using of other antivenoms available in the region and to compare their efficacy and safety with Antivipmyn-Tri in French Guiana envenomed patients.

Immunological and functional in vitro assays and preclinical efficacy of a freeze-dried antivenom manufactured in Costa Rica (Polival-ICP) against the lethal, hemorrhagic, coagulant, and myotoxic effects have recently highlighted the therapeutic potential of this antivenom against *B*. *atrox* venom endemic of French Guiana [37]. Regarding neutralization by antivenoms, Polival-ICP antivenom was effective in neutralizing lethality of *B*. *atrox* venom, while in contrast, Antivipmyn-Tri antivenom did not neutralize lethal activity even at the highest antivenom level tested [37]. In this study, the intraperitoneal route of injection was used for assessing lethality. Compared with Antivipmyn-Tri, Polival-ICP showed significantly higher neutralizing activity against hemorrhagic and in vitro coagulant activities of the venom. Polival-ICP and Antivimpyn showed similar neutralizing profile against myotoxicity [37]. These results are in agreement with previous studies showing the efficacy of Polival-ICP against the venoms of *B*. *atrox* from Colombia, Peru, Brazil, and Ecuador [71,72]. Moreover, several polyvalent antivenoms manufactured in Brazil, Argentina, Colombia, Perú, Bolivia, and Costa Rica also neutralize toxic activities of the venom of *B*. *atrox* from various localities in South America [71]. Interestingly, strong cross-reactivity of Bothrofav antivenom against *Bothrops* venoms has been shown in the neutralization of the procoagulant activity *B*. *atrox* from French Guiana [18], and this antivenom was also shown to be effective in the neutralization of other toxic activities of *B*. *atrox* venom [42]. Overall, there is an urgent need to evaluate the efficacy and safety of some of these antivenoms in the treatment of snakebite envenomation in French Guiana and to develop standard treatment protocols to be applied in this region.

### 3.2. *B*. *lanceolatus* envenomation in Martinique

In Martinique, *B*. *lanceolatus* is the only venomous snake, responsible for about 15 envenomations treated in hospitals yearly (about 3.82 per 100,000 inhabitants) [1]. The estimated mortality rate is 0.051 per 100,000 inhabitants per year [1]. *B*. *lanceolatus* venom induces local and systemic effects comparable to the typical bothropic syndrome, but the envenomation is characterized by a unique predominant prothrombotic profile. Envenomation is characterized by intense local reactions featured by extensive swelling, erythema, bleeding at the bite site, ecchymosis, and pain [3,73]. About 40% of the envenomed patients also develop systemic manifestations, such as hypo or hypertension, weakness, headache, and dyspnea [3,73]. These envenomations are often associated with systemic multifocal thrombotic complications, usually occurring within the first hours to a week after the bite, even after a moderate envenomation limited to local signs [46,74–76]. Thrombosis involves cerebral, myocardial, and pulmonary vessels and may lead to death or major functional sequelae if cases are not treated with specific antivenom [3,46,76–79]. Clinical reports indicate that anticoagulant therapy with heparin does not improve the outcomes [73,77,78]. At the same time, only rapid treatment with the monospecific commercial antivenom (Bothrofav, Sanofi-Pasteur, France) can prevent these thrombotic events [3,73].

Up to 50% of the patients envenomed by *B*. *lanceolatus* develop thrombocytopenia, while disseminated intravascular coagulation is rare [73,78,79]. Thrombocytopenia, minimally altered prothrombin time, normal activated partial thromboplastin time, and elevated fibrinogen concentration are typical features in *B*. *lanceolatus* envenomed patients who will further develop thrombosis [73,78]. The exact mechanism of thrombosis in these envenomations remains unknown, while there is not successful animal model suitable to reproduce the thrombotic manifestations [38]. As previously mentioned, a potent systemic inflammatory response [46,47], as evidenced by activation of complement products, eicosanoids release, interleukins production, and chemokines up-regulation in the human whole blood. Such inflammatory phenomena could be the main factors explaining the development of thrombotic events in *B*. *lanceolatus* envenomed patients, since such systemic inflammation in the absence of defibrinogenation might induce a procoagulant phenotype in endothelial cells.

Following the toxicological characterization of *B*. *lanceolatus* venom using various in vitro assays and in vivo mouse studies, the efficacy of monospecific Bothrofav antivenom against this venom currently in use in Martinique has been demonstrated. The monospecific antivenom, Bothrofav, manufactured by Sanofi-Pasteur, was introduced in Martinique in 1991 and has proven highly effective in preventing the development of life-threatening disturbances in these envenomations [78,80]. Experimental studies consistently showed that Bothrofav antivenom was highly effective in neutralization of all toxic and enzymatic activities of the venom of *B*. *lanceolatus* [43,81]. Third-generation antivenomics of Bothrofav antivenom confirmed the ability of this antivenom to immunocapture the toxins present in the venom of *B*. *lanceolatus*, thus underscoring the high neutralizing efficacy of this antivenom [82].

The systematic use of Bothrofav in Martinique has led to the almost complete disappearance of serious complications of *B*. *lanceolatus* envenomations and a significant reduction in thrombosis and other systemic disturbances in these envenomations [73,78,80]. However, since 2004, thromboembolic complications of the ischemic stroke type, particularly localized in the territory of the posterior and inferior cerebellar artery (PICA), have been observed in several patients after envenomation, despite a well-conducted antivenom therapy [76]. Several hypotheses were put forward to explain the reduced efficacy of the antivenom. Firstly, the activity of this antivenom developed nearly 20 years ago was reduced. Secondly, at the time of production, venoms of only few snake specimens were used to produce antivenom, which may not be representative of the variety of *B*. *lanceolatus* antigens exhibited by the venom. In line with this, WHO recommends a collection of at least 20 to 50 snakes of different sizes and geographical locations for the production of snake antivenom [83]. Hence, in response to the observed decrease in the neutralizing power of Bothrofav, a new Bothrofav2 has been marketed and used in ATU at the University Hospital of Martinique since February 2011 with a highly effective therapeutic effect [3].

Besides Bothrofav, Polival-ICP polyvalent antivenom has also been evaluated for its neutralizing ability after the characterization of venom proteomes of *B*. *lanceolatus* and *B*. *caribbaeus* snakes. Immunodepletion findings with Polival-ICP correlate well with neutralization results, since it cross reacts with *B*. *lanceolatus* SVMPs, L-amino acid oxidases, and PLA_2_s, and since a majority of venom components are recognized by antivenom antibodies in western blots and immunodepletion experiments [38]. However, since the severe thrombotic effect induced by *B*. *lanceolatus* venom has not been reproduced in the mouse model, it is not known whether Polival-ICP is able to neutralize this clinically relevant effect.

## 4. Unresolved issues and therapeutic perspectives

Several issues related to snakebite envenomation in French Guiana and Martinique remain unresolved to date and demand further experimental and clinical research. Some of these issues in Martinique are as follows: What are the pathophysiological mechanisms of envenomation by *B*. *lanceolatus*, particularly regarding the severe thrombotic effect described in human patients? Which SVMP is responsible for endothelial lesions of the microcirculation, which may lead to thrombosis? What is the role of inflammation in the thrombotic effect? What is the role of coronary angiography in case of thrombotic damage in patients suffering envenomations by *B*. *lanceolatus*? Will it be possible one day to consider the manufacture of a bivalent Bothrofav, specific for *B*. *lanceolatus* and *B*. *caribbaeus*? Would reducing the time in administering the antivenom following bites allow better control of complications? What is the benefit of using the ELISA test for assessing venom concentration in case of clinical worsening despite well-conducted antivenom therapy? What is the benefit of using metalloproteinase inhibitors in treating thrombotic stroke in these envenomations? Will Bothrofav continue to be available in Martinique?

In the case of French Guiana, some pending issues are as follows: Which is the most appropriate antivenom for the treatment of envenomations by *B*. *atrox*? What is the optimum dosage regime for antivenoms used in French Guiana? How can the time lapse between the bite and access to the hospital be reduced in French Guiana in order to avoid complications? Is it feasible to prevent local and systemic complications of these envenomations by the use of enzyme inhibitors? Would it be possible to correlate these experimental findings with the clinical observations and also with the dosage of antivenom needed in human cases?

It is therefore urgent to set up fundamental, translational, and clinical studies to better understand the mechanisms of these envenomations and treat their clinical consequences, with the identification of effective therapies: effective and safe antivenoms, antithrombotic treatments, inhibitors of the action of venom on the vascular endothelium, and aftercare active not only on *Bothrops* but also on envenomations inflicted by other tropical venomous snakes. The question of research funding by the French health authorities, with the support of international organizations such as the Pan American Health Organization (PAHO) and the World Health Organization (WHO), is therefore crucial. There is also an urgent need to develop regional collaborative and multidisciplinary approaches for the improvement of snakebite in the region and to allow access to safe, affordable antivenoms.

## 5. Concluding remarks and perspectives

Snakebite envenomation in French Guiana poses a significant challenge from the public health perspective. In the absence of a specific antivenom for French Guiana, it is necessary to undertake a detailed preclinical assessment of the various antivenoms available in Central and South America in order to identify the best option for the treatment of *B*. *atrox* envenomation in French Guiana. Moreover, protocols for the management of snakebites should be evaluated and updated, especially regarging antivenom dosage and management of complications. Another aspect that needs to be considered has to do with the availability and accessibility of antivenoms in remote rural regions and on the training of medical and nursing staff in the treatment of envenomations. Strategies for the rapid transportation of patients to health facilities need to be carefully considered, as well as the promotion of regional cooperation platforms with neighbouring countries and with regional health authorities, such as PAHO.

Although rare, envenomations by *B*. *lanceolatus* are nevertheless an important public health problem in Martinique. The clinical picture and management principles are now well established, but the search for specific therapeutics and antivenom drugs still pose unresolved problems. Preliminary clinical data available at the University Hospital of Martinique show the excellent efficacy and tolerance of an early infusion of the antivenom Bothrofav, to prevent complications of envenomation. We have thus been able to establish a harmonized protocol for managing envenomation in Martinique and include all patients in a specialized toxicology program. A joint project for Bothrofav to treat envenomations by *B*. *lanceolatus* and *B*. *caribbaeus* is also under development and will mark the beginning of a medical collaboration between the two islands, Martinique and Saint Lucia. Further studies are still needed to optimize the modalities of administration of this antivenom.

**Key Learning Points**

- Snakebite envenomation is a relevant medical hazard in French Guiana and Martinique, two French territories in the Americas.
- *B*. *atrox* is responsible for the majority of snakebites in French Guiana, while *B*. *lanceolatus* is the only venomous snake in Martinique.
- *B*. *atrox* venom typically induces coagulopathies and hemorrhage, while *B*. *lanceolatus* venom has a thrombotic effect in some patients.
- Bothrofav, a monospecific antivenom, has been successfully used in Martinique to treat envenomations.
- It is necessary to evaluate several antivenoms for their efficacy and safety in *B*. *atrox* envenomings.

**Top Five Papers**

1. Gutiérrez JM, Calvete JJ, Habib AG, Harrison RA, Williams DJ, Warrell DA. Snakebite Envenoming. Nat Rev Dis Primers. 2017;3:17063. doi: 10.1038/nrdp.2017.63
2. Resiere D, Houcke S, Pujo JM, Mayence C, Mathien C, NkontCho F, et al. Clinical Features and Management of Snakebite Envenoming in French Guiana. Toxins. 2020;12:662. doi: 10.3390/toxins12100662
3. Bourke LA, Zdenek CN, Tanaka-Azevedo AM, Silveira GPM, Sant’Anna SS, Grego KF, et al. Clinical and Evolutionary Implications of Dynamic Coagulotoxicity Divergences in Bothrops (Lancehead Pit Viper) Venoms. Toxins. 2022;14:297. doi: 10.3390/toxins14050297
4. Monteiro WM, Contreras-Bernal JC, Bisneto PF, Sachett J, Mendonça da Silva I, Lacerda M, et al. *Bothrops atrox*, the Most Important Snake Involved in Human Envenomings in the Amazon: How Venomics Contributes to the Knowledge of Snake Biology and Clinical Toxinology. Toxicon X. 2020;6:100037. doi: 10.1016/j.toxcx.2020.100037
5. Resiere D, Villalta M, Arias AS, Kallel H, Nèviére R, Vidal N, et al. Snakebite Envenoming in French Guiana: Assessment of the Preclinical Efficacy against the Venom of *Bothrops atrox* of Two Polyspecific Antivenoms. Toxicon. 2020;173:1–4. doi: 10.1016/j.toxicon.2019.11.001
